# Cross-Sectional Associations of Objectively-Measured Physical Activity and Sedentary Time with Body Composition and Cardiorespiratory Fitness in Mid-Childhood: The PANIC Study

**DOI:** 10.1007/s40279-016-0606-x

**Published:** 2016-08-24

**Authors:** Paul J. Collings, Kate Westgate, Juuso Väistö, Katrien Wijndaele, Andrew J. Atkin, Eero A. Haapala, Niina Lintu, Tomi Laitinen, Ulf Ekelund, Soren Brage, Timo A. Lakka

**Affiliations:** 1 PA Programme, MRC Epidemiology Unit, Institute of Metabolic Science, Addenbrookes Hospital, University of Cambridge, Box 285, Cambridge, CB2 0QQ UK; 2 Bradford Institute for Health Research, Bradford NHS Foundation Trust, Bradford, UK; 3 Institute of Biomedicine, Physiology, University of Eastern Finland, Kuopio, Finland; 4 Institute of Dentistry, University of Eastern Finland, Kuopio, Finland; 5 UKCRC Centre for Diet and Activity Research (CEDAR), University of Cambridge School of Clinical Medicine, Cambridge, UK; 6 Department of Biology of Physical Activity, University of Jyväskylä, Jyväskylä, Finland; 7 Department of Clinical Physiology and Nuclear Medicine, Kuopio University Hospital and University of Eastern Finland, Kuopio, Finland; 8 Department of Sport Medicine, Norwegian School of Sports Science, Oslo, Norway; 9 Kuopio Research Institute of Exercise Medicine, Kuopio, Finland

## Abstract

**Background:**

The minimum intensity of physical activity (PA) that is associated with favourable body composition and cardiorespiratory fitness (CRF) remains unknown.

**Objective:**

To investigate cross-sectional associations of PA and sedentary time (ST) with body composition and CRF in mid-childhood.

**Methods:**

PA, ST, body composition and CRF were measured in a population-based sample of 410 children (aged 7.6 ± 0.4 years). Combined heart-rate and movement sensing provided estimates of PA energy expenditure (PAEE, kJ/kg/day) and time (min/day) at multiple fine-grained metabolic equivalent (MET) levels, which were also collapsed to ST and light PA (LPA), moderate PA (MPA) and vigorous PA (VPA). Fat mass index (FMI, kg/m^2^), trunk fat mass index (TFMI, kg/m^2^) and fat-free mass index (FFMI, kg/m^2.5^) were derived from dual-energy X-ray absorptiometry. Maximal workload from a cycle ergometer test provided a measure of CRF (W/kg FFM). Linear regression and isotemporal substitution models were used to investigate associations.

**Results:**

The cumulative time above 2 METs (221 J/min/kg) was inversely associated with FMI and TFMI in both sexes (*p* < 0.001) whereas time spent above 3 METs was positively associated with CRF (*p* ≤ 0.002); CRF increased and adiposity decreased dose-dependently with increasing MET levels. ST was positively associated with FMI and TFMI (*p* < 0.001) but there were inverse associations between all PA categories (including LPA) and adiposity (*p* ≤ 0.002); the magnitude of these associations depended on the activity being displaced in isotemporal substitution models but were consistently stronger for VPA. PAEE, MPA and to a greater extent VPA, were all positively related to CRF (*p* ≤ 0.001).

**Conclusions:**

PA exceeding 2 METs is associated with lower adiposity in mid-childhood, whereas PA of 3 METs is required to benefit CRF. VPA was most beneficial for fitness and fatness, from a time-for-time perspective, but displacing any lower-for-higher intensity may be an important first-order public health strategy.

Clinical trial registry number (website): NCT01803776 (https://clinicaltrials.gov/ct2/show/NCT01803776).

## Key Points

**Table:** 

The minimum intensity of physical activity that is associated with favourable body composition and cardiorespiratory fitness remains unknown.
This study found that a higher intensity of physical activity was necessary to confer benefits to cardiorespiratory fitness (>3 metabolic equivalents) than to improve levels of total and truncal adiposity (>2 metabolic equivalents) in children aged 6–8 years, but both associations were characterised by a dose-dependent phenomenon.
Public health bodies might consider formulating recommendations purely around the concept of continuous dose-response relationships, as higher intensity physical activity may be optimal for body composition and cardiorespiratory fitness in mid-childhood, but any right-shift in the intensity distribution is likely to be beneficial.

## Introduction

Excess childhood adiposity can exert negative health effects across the life-course [1]. Preventive efforts such as increased physical activity (PA) performed during sensitive or critical time periods could help to curtail incident obesity and associated sequelae [2]. One such critical period may be mid-childhood (6–11 years), when the incidence of childhood obesity is highest [3], and when mass-specific cardiorespiratory fitness (CRF) plateaus in boys and begins to progressively decline in girls [4].

It has consistently been acknowledged that an inverse cross-sectional association exists between moderate-to-vigorous PA (MVPA) and child adiposity [5]. This is one reason why MVPA is considered an important component of paediatric obesity prevention [6] and ≥60 min MVPA/day is recommended for general childhood health [7, 8]. An additional reason is that MVPA is positively related to CRF [9], another important marker of health which seems to attenuate obesity-related co-morbidities [10, 11]. Unfortunately, few children adhere to activity recommendations [12, 13], and mid-childhood [14] and beyond [15] is epitomised by declining PA levels alongside escalating sedentary time (ST), despite guidance that ST should be minimised [7].

It remains unknown, however, if total ST is related to fat mass or fat-free mass (FFM) in young people independent of the level of MVPA [16], and due to conflicting reports it is unclear which exact PA intensities may confer benefits for body composition and CRF [17–20]. As intensity per se is a continuum, these questions need to be examined using objective exposure data which offer the required granularity to investigate the complete spectrum of objectively-measured PA intensities [21]. In particular, the lowest intensity range requires more attention. Light PA (LPA) accounts for the majority of children’s awake time [21, 22], but is often ignored [23] and consequently its merits remain largely a mystery [24]. The same applies to the effects of displacing one movement behaviour for equal time in another, which is a burgeoning line of enquiry referred to as isotemporal substitution [25]. At present, substituting ST only with MVPA has been deemed favourable for child adiposity [26, 27] and health-related fitness [27], but additional studies are needed, particularly with better characterisation of LPA. The influence of PA and ST reallocations on CRF has not yet been investigated.

The objectives of this study were to examine the entire activity intensity spectrum to establish the minimum intensities of PA that are associated with lower adiposity and higher CRF in mid-childhood, and to compare the effects of time reallocations between ST and PA intensities on these same outcomes. This information may assist the development of more palatable activity guidelines for children.

## Subjects and Methods

### Sample

This cross-sectional investigation used baseline data from the Physical Activity and Nutrition in Children (PANIC) study, a PA and diet intervention in the city of Kuopio, Finland [28]. A population-based sample of 736 children aged 6–8 years, from 16 primary schools, were invited to take part in the study. Seventy percent of children accepted (*n* = 512) and baseline measurements were conducted between October 2007 and November 2009, prior to commencement of the intervention. Participants did not differ in age, sex distribution or body mass index – standard deviation score (BMI-SDS) from all children aged 6–8 years who started first grade in the primary schools of Kuopio in 2007–2009, based on school health examination data. Altogether, 410 children (202 boys) with a mean age of 7.6 years and valid data for PA, ST, body composition and CRF were included in these analyses. All but ten children were White. All aspects of the PANIC study were approved by the Research Ethics Committee of the Hospital District of Northern Savo. Written informed consent was acquired from the parent/caregiver of each child and every child provided assent to participation.

### Assessment of Body Size and Composition

Trained staff measured children’s height (m) in the Frankfurt plane without shoes with a wall-mounted stadiometer. Body weight (kg) was measured when in light underwear by using calibrated digital scales (lnBody 720, Biospace, Seoul, Korea) after an overnight fast and after the bladder had been voided. Body mass index (kg/m^2^) was calculated and z-scores created using standard growth reference data [29]. Body fat mass (total and truncal, kg) and FFM (kg) were measured by dual energy X-ray absorptiometry (DXA, Lunar Prodigy Advance, GE Medical Systems, Madison, WI, USA) [30]. Fat mass index (FMI, kg/m^2^), trunk fat mass index (TFMI, kg/m^2^) and fat-free mass index (FFMI, kg/m^2.5^) were subsequently derived by dividing variables by height^*n*^ [31].

### Assessment of Cardiorespiratory Fitness

As described elsewhere a maximal cycle ergometer test was performed [32]. After warming up at 5 W, children cycled at a cadence of 70–80 revolutions per min for 1 min at 20 W, after which the workload steadily increased at a rate of 1 W per 6 s until voluntary exhaustion. As an indicator of CRF, peak workload was defined as the workload (Watts, W) achieved upon exercise termination, expressed relative to FFM. Heart rate was measured during the cycle test using an online ECG system (Cardiosoft v6.5 Diagnostic System, GE Healthcare Medical Systems, Freiburg, Germany) and the energy cost of the cycle ergometer protocol was derived using a subsample of participants who also had oxygen consumption measured during the test (*n* = 38). The cycle ergometer test permitted individual calibration of heart rate to physical activity energy expenditure [33].

### Assessment of Physical Activity and Sedentary Time

Subsequent to the cycle test, a combined heart rate and movement sensor (Actiheart, CamNtech Ltd, Papworth, UK) was initialised to collect data in 60 s epochs and was attached to the chest using adhesive ECG electrodes in preparation for free-living assessment. The device is lightweight and waterproof and can be worn continuously even while swimming, showering and sleeping [34]. Participants were requested to wear the monitor for a minimum of four consecutive days but some wore the monitor for up to 9 days. As school children’s activity patterns show variability between weekdays and weekends the wear period was purposefully scheduled to encompass an entire weekend [35].

Upon retrieving and downloading the combined sensor, heart rate data were first cleaned [36] then individually calibrated with parameters from the cycle test (slope, intercept and flex heart rate point) and combined with trunk acceleration in a branched equation model to estimate an intensity time-series [33]. Fourteen children did not have a valid cycle test; these children were assigned a group-level calibration derived from all valid cycle tests and represented the average heart rate to energy expenditure response for a given age, sex and sleeping heart rate. Monitor non-wear was acknowledged by prolonged zero-acceleration lasting >90 min accompanied by non-physiological heart rate, and activity estimates were adjusted during summarisation to minimise diurnal bias arising from non-wear [37]. Physical activity energy expenditure (PAEE) was calculated by integration of the intensity time-series, and the time distribution of activity intensity was generated by using standard metabolic equivalents (METs) in 0.5 increments. For these analyses, the equivalent of 5.5 ml O_2_/min/kg (110.5 J/min/kg) was used to define resting metabolic rate (1 MET) [38, 39]. Data were also collapsed to classic intensity bands; ST was defined as ≤1.5 METs, and categories of LPA (1.5–3 METs), moderate PA (MPA: >3–6 METs) and vigorous PA (VPA: >6 METs) were defined with common thresholds [24].

To derive sleep duration, a single reviewer scrutinised all activity plots to identify the timing of sleep onset (considered to be steadily declining heart rate to a persistently low level accompanied by prolonged minimal movement) and termination (abruptly increasing heart rate combined with movement onset following an extended barren period) on a day-to-day basis during overnight periods. Sleep duration (h/night) was included in analyses as a potential confounding factor [40]. To separate sleep and ST, the average daily sleep duration was subtracted from the average daily time in ≤1.5 METs.

Valid PA was defined as records containing ≥48-h data, with ≥32 and ≥16 observed weekday and weekend hours, respectively. It was further required that data were represented by ≥12 h of morning, noon, afternoon and evening wear time. This caveat regarding the time-distribution of observed data shielded against bias from over-representation of specific parts of days and optimised the diurnal bias minimisation procedure [41]. The proportion of total wear arising from weekends and the timing (season) of activity measurements was captured.

### Other Assessments

Parents reported their child’s age, sex, birth weight and the household income of the highest earner in euros; participants were classified as belonging to low (<30,000 €), middle (30,000 to <60,000 €) or high (≥60,000 €) annual income families. Parents reported their weight (kg) and height (m) at the baseline assessment and parental BMI was calculated. Following detailed instruction from a nutritionist, parents completed a 4-day food record for their child. The food record included an entire weekend and nutrient intakes were calculated using the Micro Nutrica^®^ dietary analysis program, version 2.5 (Social Insurance Institution of Finland, Turku, Finland) with recent updates in the nutrition composition database. For this specific analysis, total energy intake (kJ/day) and fat intake (g/day) were regarded as potentially important confounding factors. Further information regarding eating pattern was collected with the parent-reported Children’s Eating Behaviour Questionnaire, which has been validated in diverse groups [42, 43]. Children were classified as ‘every day’ or ‘irregular’ breakfast consumers, eating ‘three’ or ‘fewer than three’ main meals daily, and according to their snacking habit (<2, 2–3 or >3 snacks daily).

### Statistics

This investigation was restricted to participants with valid data for PA, ST, body composition and CRF. To describe the sample, summary statistics were calculated (mean ± standard deviation for normal distributions, median (inter-quartile range) for skewed distributions, and percentages for categories). Sex comparisons were made using linear (continuous variables) or logistic (categorical variables) regression accounting for school clustering with robust standard errors. Spearman’s correlations were calculated between all activity categories. To compare the characteristics of contributing children to non-contributors (excluded due to missing exposure and/or outcome data), linear or logistic regression with robust standard errors was used, adjusted for sex and age when these were not the variables of interest.

To allow for missing data in some covariates for 77 children (19 % of the sample; mainly parental BMI was missing), multiple imputation by chained equations was used to investigate associations of PA and ST with body composition and CRF. Ten imputed datasets were created and linear regression analysis performed, again using robust standard errors [44]. Crude models were initially analysed, as were minimally adjusted models controlling only for monitor wear characteristics (the proportion of weekend data and season of measurement). Adjustment for demographic variables was subsequently made (age, sex, household income; ethnicity was not included due to low variation) followed by adjustment for behaviours (sleep duration, energy intake, frequency of breakfast consumption, number of meals per day, snacking). In a further model, birth weight and parental BMI were included. Lastly, for models with body composition as outcomes, adjustment for CRF was made, and models with CRF as the outcome were adjusted for FMI.

The above described models were first used to investigate associations of the cumulative time (min/day) above single MET activity intensities (that is the total time spent in activity >1 MET, >2 METs, >3 METs and so on up to >7 METs; each intensity occupied a single model) with FMI, TFMI, FFMI and CRF. Models were then used to investigate associations of the broader ST and PA intensity bands and PAEE with the same outcomes. In this second analysis two primary models were constructed. The first was adjusted for all aforementioned factors added to models in the order already described (monitor wear characteristics followed by demographics, behavioral factors, birth weight, parental BMI, FMI or CRF). The second model (not applicable to PAEE) was built in the same manner, but from the outset mutual adjustment for each of the PA intensities was performed by simultaneously including LPA, MPA and VPA in the linear predictor. The results for this second model, which is constrained to the non-variant 24-h per day by also adjusting for sleep duration and leaving out only ST, represent isotemporal substitution; the effect on the outcome of exchanging a unit of ST for PA (inverting the results represents the effect of exchanging a unit of PA for ST). To cover all isotemporal eventualities the omitted variable alternated across all activity intensities [25]. All results have been scaled to represent the association between exposures and outcomes per 10 unit (min/day; or kJ/kg/day for PAEE) difference in exposures. Owing to skewed distributions, FMI and TFMI were natural log-transformed prior to analyses and have been back-transformed to represent the percentage difference in outcomes [formula = ((exp(β × 10) − 1) × 100)]. All models were tested for effect modification by sex. Plots of residuals were reviewed and multicollinearity was checked by variance inflation factors. Statistical analyses were conducted in Stata/SE 13.1 (StataCorp, College Station, TX, USA). Results with *p* values <0.05 were deemed statistically significant.

## Results

### Sample Description

Table 1 describes the 410 participants (49 % boys) with valid data for PA, ST, body composition and CRF. Girls weighed less and were shorter, and had lower birth weight and energy and fat intake than boys. Non-participants (*n* = 102) were characterised by a greater proportion of boys (61.8 %, *p* = 0.024) and were older (7.7 vs. 7.6 years, *p* = 0.042) compared to children who contributed to the analysis.

**Table 1:** Characteristics of study participants

Parameter	Boys (*n* = 202)		Girls (*n* = 208)		*p* for sex difference
	*n*	Value	*n*	Value	
Age (y)	202	7.7 ± 0.4^a^	208	7.6 ± 0.4	0.089
Ethnicity (*n* (%) White)	202	197 (97.5)	208	203 (97.6)	0.96
Household income (*n* (%))	194		206		
Low	**–**	37 (19.1)	–	45 (21.8)	
Middle	**–**	76 (39.2)	–	92 (44.7)	
High	**–**	81 (41.8)	–	69 (33.5)	0.14
Weight (kg)	202	26.9 (5.6)^*b*^	208	25.3 (5.8)	0.030
Height (cm)	202	130.0 ± 5.4	208	127.6 ± 5.5	0.002
BMI (kg/m^2^)	202	15.8 (2.4)	208	15.6 (2.2)	0.52
BMI *z* score^*c*^	202	0.092 ± 1.2	208	−0.078 ± 1.0	0.19
Birth weight (g)	199	3628 ± 565	205	3475 ± 533	0.018
Sleep duration (h/night)	202	9.6 ± 0.5	208	9.7 ± 0.5	0.45
Breakfast (*n* (%) every day)	197	166 (84.3)	204	179 (87.7)	0.42
Daily meals (*n* (%) three/d)	197	83 (42.1)	204	75 (36.8)	0.37
Daily snacking (*n* (%))	197		204		
<2 snacks	**–**	25 (12.7)	–	31 (15.2)	
2–3 snacks	**–**	128 (65.0)	–	117 (57.4)	
>3 snacks	**–**	44 (22.3)	–	56 (27.5)	0.63
Energy intake (kJ/day)	197	7205 ± 1314	204	6423 ± 1154	<0.001
Fat intake (g/day)	197	58 ± 15	204	51 ± 14	0.002
Maternal BMI (kg/m^2^)	187	23.5 (5.0)	201	23.7 (4.7)	0.84
Paternal BMI (kg/m^2^)	181	26.2 (4.1)	186	25.8 (4.9)	0.32

*BMI* body mass index

^a^Mean and standard deviation for all such values with normal distribution

^b^Median and inter-quartile range in parentheses for all such values with skewed distribution

^c^Based on British growth reference data; sex-comparisons performed using linear regression for continuous variables (variables with skewed distribution were natural log-transformed prior to analyses), logistic regression for categorical variables with 2-levels, and ordered logistic regression for categorical variables with >2 levels, all adjusted for school clustering by robust standard errors

**Fig. 1:**
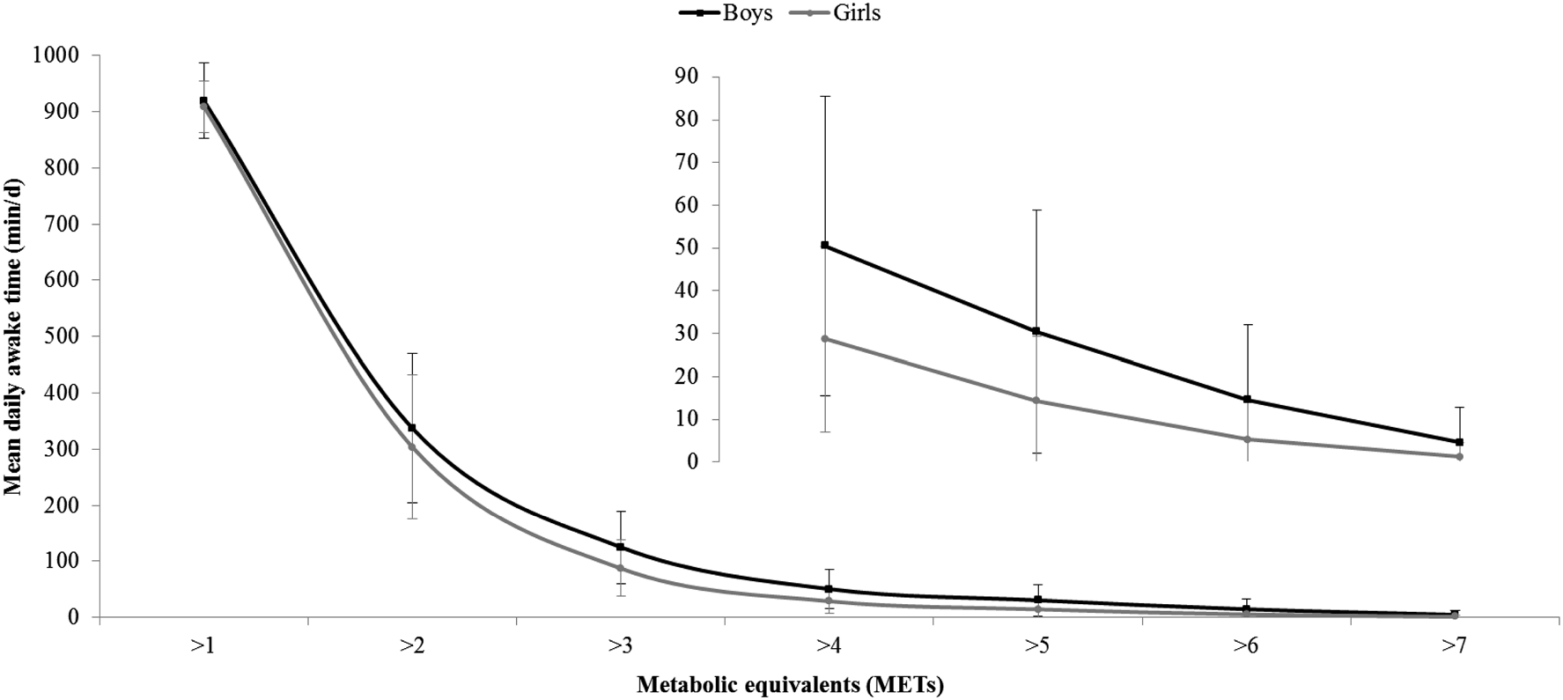
Daily cumulative awake time spent above single-MET categories. Data are mean values and error bars represent ± standard deviation. Sedentary time corresponds to the region: ≤1.5 METs; light physical activity: 1.5–3 METs; moderate physical activity: >3–6 METs; vigorous physical activity: >6 METs. *Inset* shows magnified plot for >4 METs. *METs* metabolic equivalents

Table 2 provides summary data for PA, ST, body composition and CRF. The mean (± standard deviation) duration of monitor wear was 111.8 (± 32.5) hours, equivalent to 4.7 (± 1.4) full days, and one-third (32.5 ± 12.1 %) of all data were from weekends; monitor non-wear was minimal with an average 1438 (± 4.6) min/day of data registered by devices from a possible 1440 min/day. Compared to boys, girls were characterised by higher levels of LPA, FMI and TFMI, but lower levels of higher-intensity PA (≥MPA), PAEE, FFMI and CRF. Sex differences remained after adjusting CRF for the season of assessment, and adjusting PA and ST variables for the proportion of weekend data and season. Figure 1 provides a pictorial representation of the cumulative intensity distribution during awake time, showing an exponential decline in time as a function of intensity. Spearman’s analysis showed that the strongest correlation for time-based exposures was between ST and LPA (*r* = −0.76, *p* < 0.001). MPA and VPA were also strongly correlated (*r* = 0.68, *p* < 0.001), as were ST (*r* = −0.83), LPA (*r* = 0.43), MPA (*r* = 0.92) and VPA (*r* = 0.71) with PAEE (*p* < 0.001 for all).

**Table 2:** Distributions of sedentary time, physical activity, body composition and cardiorespiratory fitness

Parameter	Boys (*n* = 202)	Girls (*n* = 208)	*p* for sex difference
Sedentary time (min/day)	367.2 ± 114.4^a^	372.4 ± 118.4	0.69
Light PA (min/day)	370.6 ± 83.4	400.3 ± 93.4	0.035
Moderate PA (min/day)	102.3 (76.2)^*b*^	74.4 (59.1)	<0.001
Vigorous PA (min/day)	8.6 (20.5)	1.9 (6.3)	<0.001
PAEE (kJ/kg/day)	99.3 ± 31.4	85.5 ± 25.9	0.001
Fat mass index (kg/m^2^)	2.4 (2.0)	3.2 (1.9)	<0.001
Trunk fat mass index (kg/m^2^)	0.9 (0.8)	1.3 (0.9)	0.001
Fat-free mass index (kg/m^2.5^)	11.3 ± 0.7	10.5 ± 0.7	<0.001
CRF (W/kg FFM)	3.8 ± 0.5	3.6 ± 0.5	<0.001

*CRF* cardiorespiratory fitness, *FFM* fat-free mass, *PA* physical activity, *PAEE* physical activity energy expenditure

^a^Mean and standard deviation for all such values with normal distribution

^b^Median and inter-quartile range in parentheses for all such values with skewed distribution; sex-comparisons performed using linear regression adjusted for school clustering by robust standard errors (variables with skewed distribution were natural log-transformed prior to analyses). Sedentary time: ≤1.5 METs; Light PA: 1.5–3 METs; Moderate PA: >3–6 METs; Vigorous PA: >6 METs. PA variables were individually calibrated to cycle test heart rate response in all but 14 children, who were assigned the average response of the group: PAEE (J/min/kg) = (7.65 − 0.0063 × age + 0.59 × sex − 0.0014 × SHR) × HRaS − 1.08 × age − 9.55 × sex + 0.58 × SHR + 0.040 × SHR × sex − 190.69 (age in years, sex coded as 1 for boys and 0 for girls, SHR is sleeping heart rate in beats per minute, HRaS is heart rate above SHR in beats per minute, FlexHRaS = −0.16 × SHR + 48)

### Cumulative Intensity Analyses

**Fig. 2:**
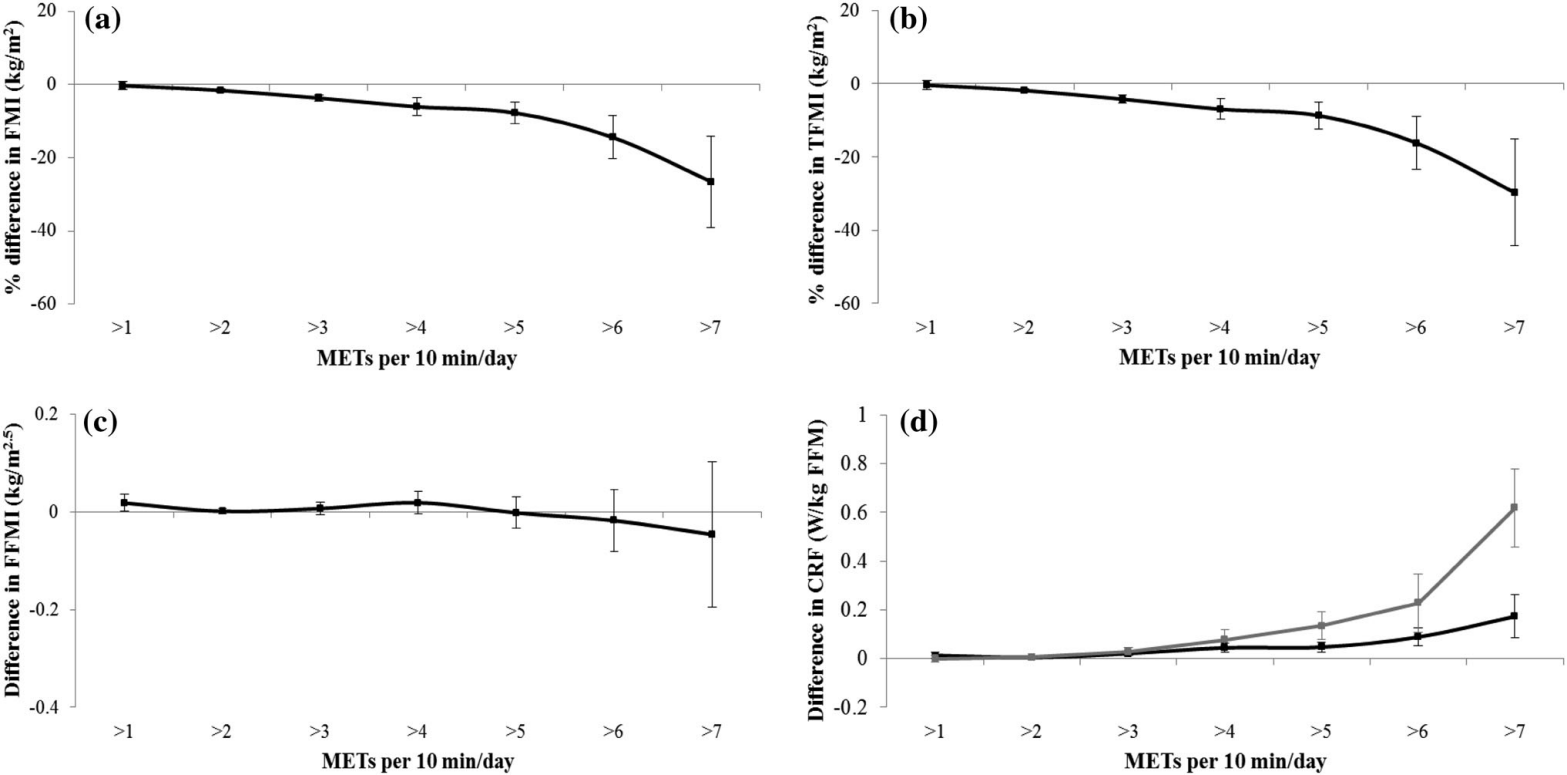
Associations between the cumulative awake time above single MET categories and **a** FMI, **b** TFMI, **c** FFMI and **d** CRF. Statistical analyses performed using multiple imputed datasets and linear regression adjusted for physical activity monitor wear characteristics (proportion of weekend data and season of measurement), demographics (age, sex, household income), behaviours (sleep duration, energy intake, frequency of breakfast consumption, number of meals per day, snacking), birth weight, maternal and paternal BMI. Adjustment for CRF was further made when FMI, TFMI and FFMI were outcomes and CRF was adjusted for FMI. School clustering was accounted for by using robust standard errors. FMI and TFMI were skewed and natural log-transformed prior to analyses, their data have been back-transformed to represent the percentage difference (95 % CI) in variables per 10 min spent above a MET level. All results are scaled to represent the association between exposures and outcomes per 10 min difference in exposures. *METs* metabolic equivalents, *FMI* fat mass index, *TFMI* trunk fat mass index, *FFMI* fat-free mass index, *CRF* cardiorespiratory fitness

With the exception of CRF, there was no consistent evidence for sex-interactions of the associations between cumulative intensity distributions and outcomes. Figure 2 therefore shows associations for body composition that are based on the whole sample, whereas results for CRF are stratified by sex. Associations are adjusted for covariates, including mutual adjustment for body composition and CRF which did not alter the results. Time spent above 2 METs was inversely associated with FMI and TFMI (Fig. 2a, b; *p* ≤ 0.006) but there was no consistent evidence for associations between MET values and FFMI (Fig. 2c). In Fig. 2d, the cumulative time above 3 METs was positively associated with CRF in boys and girls (*p* ≤ 0.002 for both). The associations for FMI, TFMI and CRF were all characterised by a graded dose-response relationship with the greatest returns achieved from increasing activity intensity beyond 7 METs; every 10 min of PA >7 METs was associated with approximately 26–30 % lower FMI and TFMI, and a higher CRF of 0.17 and 0.62 W/kg FFM in boys and girls, respectively (*p* ≤ 0.006 for all).

### Categorical Analyses and Isotemporal Substitution Models

Associations between classical ST and PA categories with outcomes are shown in Table 3. There was no consistent evidence of sex-interactions. In Model 1, independent of covariates including CRF, ST was positively associated, and LPA, MPA and VPA were all significantly inversely associated, with adiposity; the magnitude of associations were largely equivalent for FMI and TFMI, and strongest for VPA. PAEE was also inversely associated with adiposity. Regardless of adjustment for CRF there was no indication that any activity parameter was related to FFMI. There were also no associations between ST and LPA with CRF. Conversely, MPA and VPA displayed positive associations with CRF, and the association was strongest for VPA. PAEE was also positively associated with CRF, independent of adiposity. The isotemporal substitution results (Model 2) revealed that substituting ST with LPA or MPA was inversely associated with FMI and TFMI, but if the same amount of time was instead shifted from ST to VPA, the association was much stronger; exchanging 10 min ST/day for VPA/day equated to approximately 12–13 % lower total and truncal fatness. Swapping LPA or MPA with VPA was also significantly inversely associated with FMI and TFMI, to approximately the same magnitudes. Consistent with Model 1 there were no significant associations for FFMI. With respect to CRF, there was no evidence of an association for substituting ST with LPA, but exchanging ST or LPA with MPA was positively associated with CRF. The greatest impacts on CRF, however, came from shifting ST, LPA and MPA into VPA.

**Table 3:** Associations of categories for sedentary time and physical activity with body composition and cardiorespiratory fitness

	FMI (% difference, kg/m^2^)		TFMI (% difference, kg/m^2^)		FFMI (kg/m^2.5^)		CRF (W/kg FFM)	
	*β* (95% CI)	*p*	*β* (95% CI)	*p*	*β* (95% CI)	*p*	*β* (95% CI)	*p*
Model 1								
Sedentary time	**1.8 (1.3 to 2.3)**	**<0.001**	**2.1 (1.5 to 2.7)**	**<0.001**	−0.0013 (−0.0085 to 0.0059)	0.71	−0.0039 (−0.0083 to 0.00063)	0.086
Light PA	**−1.6 (−2.2 to −1.0)**	**<0.001**	**−1.9 (−2.6 to −1.2)**	**<0.001**	−0.0004 (−0.010 to 0.0095)	0.93	−0.0038 (−0.010 to 0.0024)	0.21
Moderate PA	**−3.7 (−4.7 to −2.7)**	**0.002**	**−4.3 (−5.5 to −3.0)**	**<0.001**	0.0097 (−0.0051 to 0.025)	0.18	**0.023 (0.015 to 0.031)**	**<0.001**
Vigorous PA	**−14.4 (−20.3 to −8.2)**	**<0.001**	**−16.2 (−23.4 to −8.4)**	**0.001**	−0.018 (−0.082 to 0.045)	0.55	**0.12 (0.059 to 0.18)**	**0.001**
PAEE	**−8.0 (−9.8 to −6.2)**	**<0.001**	**−9.2 (−11.4 to −6.9)**	**<0.001**	0.0087 (−0.017 to 0.034)	0.48	**0.046 (0.027 to 0.064)**	**<0.001**
Model 2								
ST → light PA	**−1.2 (−1.8 to −0.64)**	**0.001**	**−1.5 (−2.1 to −0.82)**	**<0.001**	−0.0026 (−0.013 to 0.0079)	0.61	−0.0038 (−0.010 to 0.0027)	0.23
ST → moderate PA	**−1.7 (−2.7 to −0.78)**	**0.002**	**−2.0 (−3.2 to −0.83)**	**0.003**	0.016 (−0.0002 to 0.031)	0.052	**0.014 (0.0064 to 0.022)**	**0.002**
ST → vigorous PA	**−11.8 (−17.4 to −5.8)**	**0.001**	**−13.1 (−20.1 to −5.5)**	**0.003**	−0.042 (−0.12 to 0.033)	0.25	**0.098 (0.040 to 0.16)**	**0.003**
Light → moderate PA	−0.51 (−1.7 to 0.73)	0.39	−0.58 (−2.0 to 0.86)	0.40	0.018 (−0.0042 to 0.041)	0.10	**0.018 (0.0054 to 0.031)**	**0.009**
Light → vigorous PA	**−10.7 (−16.2 to −4.8)**	**0.002**	**−11.8 (−18.8 to −4.3)**	**0.005**	−0.039 (−0.11 to 0.034)	0.27	**0.10 (0.046 to 0.16)**	**0.002**
Moderate → vigorous PA	**−10.2 (−16.4 to −3.5)**	**0.006**	**−11.3 (−19.0 to −2.9)**	**0.013**	−0.057 (−0.14 to 0.024)	0.15	**0.083 (0.024 to 0.14)**	**0.01**

Statistical analyses performed using multiple imputed datasets and linear regression adjusted for PA monitor wear characteristics (proportion of weekend data and season of measurement), demographics (age, sex, household income), behaviours (sleep duration, energy intake, frequency of breakfast consumption, number of meals per day, snacking), birth weight, maternal and paternal BMI. Adjustment for CRF was further made when FMI, TFMI and FFMI were outcomes and CRF was adjusted for FMI. School clustering was accounted for by using robust standard errors. FMI and TFMI were skewed and natural log-transformed prior to analyses, their data have been back-transformed to represent the percentage difference in variables. All results are scaled to represent the association between exposures and outcomes per 10 unit difference in exposures and statistically significant associations are in bold. Model 2 shows isotemporal substitution results and the effect of exchanging 10 min of ST or PA for different PA intensities. For example, shifting 10 min from ST to light PA was associated with 1.2 % lower FMI. Sedentary time: ≤1.5 METs; Light PA: 1.5–3 METs; Moderate PA: >3–6 METs; Vigorous PA: >6 METs

*ST* sedentary time, *PA* physical activity, *PAEE* physical activity energy expenditure, *FMI* fat mass index, *TFMI* trunk fat mass index, *FFMI* fat-free mass index, *CRF* cardiorespiratory fitness

All results were materially similar when adjusted for sex and age and without these adjustments, thereby providing reassurance that results were not biased by exclusion of participants with missing data for PA, ST, body composition or CRF. Results were also materially unchanged from complete-case analyses (*n* = 333) and when adjustment for fat intake was performed instead of total energy intake. Residual plots showed no strong evidence of heteroscedasticity and all variance inflation factors were well within tolerance (≤3.2).

## Discussion

This study of 410 Finnish children aged 6–8 years has replicated the consistent cross-sectional finding of an inverse association between MVPA and child adiposity [5, 16]. However, by investigating the spectrum of intensities, we uniquely found that exceeding an intensity of at least 2 METs (i.e. a PA intensity >110.5 J/min/kg above resting) was inversely associated with DXA-derived FMI and TFMI. Accompanying this observation, exchanging daily ST for LPA was associated with lower total and truncal adiposity. Nonetheless, higher-intensity activity conferred greater benefit per unit time, as has been shown elsewhere [19]. Isotemporal substitution models anchored on ST showed that for equivalent reductions in adiposity the time requirement for VPA was 7–10 times shorter than LPA and MPA. Likewise, to attain equivalent gains in CRF, sevenfold less VPA than MPA, substituted for ST, was needed.

We have previously shown in a UK cohort of adolescents that LPA can substantially contribute to PAEE [21], but its association with child adiposity has remained largely equivocal. Some studies have reported inverse associations between objectively-measured LPA and markers of childhood fatness [45–47]. Others have reported no such relation [19, 20, 26, 27, 48–51]. These inconsistencies may simply relate to how LPA has been defined, but it is noteworthy that most studies reporting an inverse association for LPA have measured fatness by DXA, whereas studies with null results have more frequently used proxy measures such as BMI or waist circumference, therefore measurement error may account for some of the null findings [52]. Our data suggest that adequate LPA may be an effective, but not optimal, means of maintaining total and truncal adiposity levels and offsetting unhealthy fat gain in mid-childhood. Importantly, the data further highlight that LPA is accessible regardless of FFMI and CRF levels; we found no association between LPA and any other activity parameter with FFMI (as shown previously in pre-schoolers [51]) and LPA was not associated with CRF which concurs with results from elsewhere [19, 20, 46].

Contrasting the results for LPA, we observed that MPA and VPA were both inversely associated with adiposity and also positively associated with CRF, with the magnitude of associations being largest for VPA. In agreement we found that the cumulative time above 3 METs was positively associated with CRF in a dose-dependent manner. Our data partially concur with those of others, who similarly found that MPA and VPA were both positively associated with CRF, but concluded that only VPA was associated with body fatness [17–19]. Our results indicate that MPA, and particularly VPA within a restricted time budget, may be the optimal intensity domains for improving adiposity and CRF levels in children. We also found that PAEE was inversely associated with FMI and TFMI, and positively related to CRF, which raises the question whether PAEE may mediate the reported associations of MPA and VPA with adiposity or CRF. Given the compositional nature of PAEE being made up of all intensities, both MPA and VPA were strongly positively correlated with PAEE, which made it inappropriate to mutually adjust for these parameters in analyses. Under the naïve assumption of everything else being equal (most notably energy intake) it is conceivable that PA volume may be the decisive factor for body composition and not intensity per se. Nevertheless, there are many biologically plausible arguments as to why activity intensity may be important for adiposity over and above energy expenditure, including appetite regulation and the lag-effect of increased post-activity resting metabolism [51]. For these reasons, future work should try to clarify if activity intensity is related to adiposity independent of PA volume. Potentially this could be achieved by estimating the substitution effect of energy expended at one intensity level for energy expended at another intensity [26]. With regard to CRF, there is increasing acceptance that only MVPA, and in particular VPA, can incite improvements [17–20].

Categorical analyses (Table 3) revealed that ST was positively associated with total and truncal adiposity in both sexes. Isotemporal substitution models further revealed that replacing ST with an equal volume of LPA or MPA, and more so VPA, was beneficial for body composition. This contrasts reports based on awake-time accelerometry which indicate that replacing ST only with MVPA is favourable for childhood adiposity [26, 27]. The difference may be that the current study utilised uninterrupted (24-h/day) combined heart rate and movement sensing to better characterise ST and all categories of PA, including LPA. For CRF there were null effects of substituting ST with LPA, but replacing ST or LPA with time-equivalent MPA was positively associated with CRF, and the magnitude of association was larger if time was substituted for VPA. Therefore, VPA seems to confer the most benefit for fitness on the basis of like-for-like time displacement with ST or other PA intensities. It should nevertheless be considered that displacing ST for VPA, for example, would likely constitute a challenging public health proposal given the current obesity and inactivity pandemics, which are set inside what has been termed a ‘slothogenic’ environment and society [53]. Potentially a more reasonable and achievable first goal would be to focus on displacing ST in favour of any PA intensity for lower adiposity, whilst emphasising the greater returns offered by higher-intensity PA (of at least moderate and particularly vigorous intensity), such as further improved body composition and elevated CRF. The results for adiposity and CRF were independent of one another, implying that the reported benefits of PA for weight management and fitness would likely extend widely to children regardless of these factors.

Strengths of this study include the population-based sample of children, a maximal cycle ergometer test for CRF evaluation, combined-sensing estimates of ST and PA with individual calibration of heart rate, and measurement of body composition by DXA. Many studies have used DXA as a criterion standard and it is a superior technique to field-based methods [54]. Combined-sensing also outperforms accelerometry or heart-rate alone in estimating PAEE [37, 55, 56] and low-to-moderate PA [57]. It is unfortunate, nonetheless, that a relatively short observation period was used, meaning that we might not have captured representative ST and PA profiles for all children. This could have biased the reported associations toward the null, suggesting that the true associations may be even stronger than we report. Although we controlled for many variables that were plausibly related to exposures and outcomes (including dietary consumption, eating patterns, and sleep duration, which is recommended [26] but rarely achieved) residual confounding is a potential issue in all observational studies. It is also a weakness that the direction of association between variables is indeterminable due to the cross-sectional study design. This is particularly problematic because bidirectional associations may exist between exposures and outcomes; thus our results may equally imply that fitter and less fat children have more favourable ST and PA profiles. Longitudinal studies are needed in larger and more representative samples.

## Conclusion

This study found that a higher intensity of PA was necessary to confer benefits to CRF (>3 METs) than to improve body composition (>2 METs), but both associations were ultimately characterised by a dose-dependent phenomenon. It therefore seems that LPA can benefit child body composition but at least moderate intensity PA is required for higher fitness. Vigorous PA will provide the greatest time investment returns for both fitness and fatness, but ST should not be ignored as it was positively associated with both total and truncal adiposity. It seems that a pertinent starting point for public health bodies would be to formulate recommendations purely around the concept of dose-dependent relationships; health benefits can be derived from higher doses of PA, achieved either through longer duration or higher intensity or both. If time is a limiting factor, activity at higher intensity offers an efficient intervention strategy but any right-shift in the intensity distribution is likely to be beneficial.

## Abbreviations

BMI: Body mass index
CRF: Cardiorespiratory fitness
DXA: Dual-energy X-ray absorptiometry
FMI: Fat mass index
FFM: Fat-free mass
FFMI: Fat-free mass index
LPA: Light physical activity
METs: Metabolic equivalents
MPA: Moderate physical activity
MVPA: Moderate-to-vigorous physical activity
PA: Physical activity
PAEE: Physical activity energy expenditure
ST: Sedentary time
TFMI: Trunk fat mass index
VPA: Vigorous physical activity

## References

[1] Reilly JJ, Kelly J (2011). Long-term impact of overweight and obesity in childhood and adolescence on morbidity and premature mortality in adulthood: systematic review. Int J Obes..

[2] Dietz WH (1994). Critical periods in childhood for the development of obesity. Am J Clin Nutr..

[3] Hughes AR, Sherriff A, Lawlor DA, Ness AR, Reilly JJ (2011). Incidence of obesity during childhood and adolescence in a large contemporary cohort. Prevent Med..

[4] Armstrong N (2013). Aerobic fitness and physical activity in children. Pediatr Exerc Sci..

[5] Jimenez-Pavon D, Kelly J, Reilly JJ (2010). Associations between objectively measured habitual physical activity and adiposity in children and adolescents: systematic review. Int J Pediatr Obes..

[6] Graf C (2011). Preventing and treating obesity in pediatrics through physical activity. EPMA J..

[7] Department of Health, Physical Activity, Health Improvement and Protection. Start active, stay active: a report on physical activity from the four home countries’ Chief Medical Officers. London: Department of Health; 2011.

[8] World Health Organization (WHO) (2010). Global recommendations on physical activity for health.

[9] Armstrong N, Tomkinson GR, Ekelund U (2011). Aerobic fitness and its relationship to sport, exercise training and habitual physical activity during youth. Br J Sport Med..

[10] Ortega FB, Ruiz JR, Castillo MJ, Sjostrom M (2008). Physical fitness in childhood and adolescence: a powerful marker of health. Int J Obes..

[11] DuBose KD, Eisenmann JC, Donnelly JE (2007). Aerobic fitness attenuates the metabolic syndrome score in normal-weight, at-risk-for-overweight, and overweight children. Pediatrics..

[12] Griffiths LJ, Cortina-Borja M, Sera F, Pouliou T, Geraci M, Rich C (2013). How active are our children? Findings from the Millennium Cohort Study. BMJ Open..

[13] Hallal PC, Andersen LB, Bull FC, Guthold R, Haskell W, Ekelund U (2012). Global physical activity levels: surveillance progress, pitfalls, and prospects. Lancet..

[14] Dumith SC, Gigante DP, Domingues MR, Kohl HW (2011). Physical activity change during adolescence: a systematic review and a pooled analysis. Int J Epidemiol..

[15] Collings PJ, Wijndaele K, Corder K, Westgate K, Ridgway CL, Sharp SJ (2015). Magnitude and determinants of change in objectively-measured physical activity, sedentary time and sleep duration from ages 15 to 17.5 y in UK adolescents: the ROOTS study. Int J Behav Nutr Phys Act..

[16] Ekelund U, Hildebrand M, Collings PJ (2014). Physical activity, sedentary time and adiposity during the first two decades of life. Proc Nutr Soc..

[17] Ruiz JR, Rizzo NS, Hurtig-Wennlof A, Ortega FB, Warnberg J, Sjostrom M (2006). Relations of total physical activity and intensity to fitness and fatness in children: the European Youth Heart Study. Am J Clin Nutr..

[18] Gutin B, Yin Z, Humphries MC, Barbeau P (2005). Relations of moderate and vigorous physical activity to fitness and fatness in adolescents. Am J Clin Nutr..

[19] Hay J, Maximova K, Durksen A, Carson V, Rinaldi RL, Torrance B (2012). Physical activity intensity and cardiometabolic risk in youth. Arch Pediatr Adolesc Med..

[20] Aires L, Silva P, Silva G, Santos MP, Ribeiro JC, Mota J (2010). Intensity of physical activity, cardiorespiratory fitness, and body mass index in youth. J Phys Act Health..

[21] Collings PJ, Wijndaele K, Corder K, Westgate K, Ridgway CL, Dunn V (2014). Levels and patterns of objectively-measured physical activity volume and intensity distribution in UK adolescents: the ROOTS study. Int J Behav Nutr Phys Act..

[22] Hesketh KR, McMinn AM, Ekelund U, Sharp SJ, Collings PJ, Harvey NC (2014). Objectively measured physical activity in four-year-old British children: a cross-sectional analysis of activity patterns segmented across the day. Int J Behav Nutr Phys Act..

[23] Katzmarzyk PT (2010). Physical activity, sedentary behavior, and health: paradigm paralysis or paradigm shift?. Diabetes..

[24] Janssen I, LeBlanc AG (2010). Systematic review of the health benefits of physical activity and fitness in school-aged children and youth. Int J Behav Nutr Phys..

[25] Mekary RA, Willett WC, Hu FB, Ding EL (2009). Isotemporal substitution paradigm for physical activity epidemiology and weight change. Am J Epidemiol..

[26] Loprinzi PD, Cardinal BJ, Lee H, Tudor-Locke C (2015). Markers of adiposity among children and adolescents: implications of the isotemporal substitution paradigm with sedentary behavior and physical activity patterns. J Diabetes Metabol Disord..

[27] Aggio D, Smith L, Hamer M (2015). Effects of reallocating time in different activity intensities on health and fitness: a cross sectional study. Int J Behav Nutr Phy..

[28] Eloranta AM, Lindi V, Schwab U, Tompuri T, Kiiskinen S, Lakka HM (2012). Dietary factors associated with overweight and body adiposity in Finnish children aged 6-8 years: the PANIC Study. Int J Obes..

[29] Cole TJ, Freeman JV, Preece MA (1995). Body mass index reference curves for the UK, 1990. Arch Dis Child..

[30] Tompuri TT, Lakka TA, Hakulinen M, Lindi V, Laaksonen DE, Kilpelainen TO (2015). Assessment of body composition by dual-energy X-ray absorptiometry, bioimpedance analysis and anthropometrics in children: the Physical Activity and Nutrition in Children study. Clin Physiol Funct Imaging..

[31] Wells JC (2001). A critique of the expression of paediatric body composition data. Arch Dis Child..

[32] Lintu N, Tompuri T, Viitasalo A, Soininen S, Laitinen T, Savonen K (2014). Cardiovascular fitness and haemodynamic responses to maximal cycle ergometer exercise test in children 6–8 years of age. J Sport Sci..

[33] Brage S, Ekelund U, Brage N, Hennings MA, Froberg K, Franks PW (2007). Hierarchy of individual calibration levels for heart rate and accelerometry to measure physical activity. J Appl Physiol..

[34] Brage S, Brage N, Franks PW, Ekelund U, Wareham NJ (2005). Reliability and validity of the combined heart rate and movement sensor Actiheart. Eur J Clin Nutr..

[35] Brooke HL, Corder K, Atkin AJ, van Sluijs EMF (2014). A systematic literature review with meta-analyses of within- and between-day differences in objectively measured physical activity in school-aged children. Sports Med..

[36] Stegle O, Fallert SV, MacKay DJC, Brage S (2008). Gaussian process robust regression for noisy heart rate data. IEEE Trans Bio Med Eng..

[37] Brage S, Westgate K, Franks PW, Stegle O, Wright A, Ekelund U (2015). Estimation of free-living energy expenditure by heart rate and movement sensing: a doubly-labelled water study. PloS One..

[38] Henry CJ (2005). Basal metabolic rate studies in humans: measurement and development of new equations. Public Health Nutr..

[39] Evenson KR, Catellier DJ, Gill K, Ondrak KS, McMurray RG (2008). Calibration of two objective measures of physical activity for children. J Sports Sci..

[40] Collings PJ, Wijndaele K, Corder K, Westgate K, Ridgway CL, Sharp SJ (2015). Prospective associations between sedentary time, sleep duration and adiposity in adolescents. Sleep Med..

[41] Brage S, Westgate K, Wijndaele K, Godinho J, Griffin S, Wareham N. Evaluation of a method for minimising diurnal information bias in objective sensor data. Int Conf Amb Mon Phys Act Mov. 2013(Conference Proceeding).

[42] Carnell S, Wardle J (2007). Measuring behavioural susceptibility to obesity: validation of the child eating behaviour questionnaire. Appetite..

[43] Viana V, Sinde S, Saxton JC (2008). Children’s Eating Behaviour Questionnaire: associations with BMI in Portuguese children. Br J Nutr..

[44] White IR, Royston P, Wood AM (2011). Multiple imputation using chained equations: issues and guidance for practice. Stat Med..

[45] Treuth MS, Hou N, Young DR, Maynard LM (2005). Accelerometry-measured activity or sedentary time and overweight in rural boys and girls. Obes Res..

[46] Butte NF, Puyau MR, Adolph AL, Vohra FA, Zakeri I (2007). Physical activity in nonoverweight and overweight Hispanic children and adolescents. Med Sci Sports Exerc..

[47] Kwon S, Janz KF, Burns TL, Levy SM (2011). Association between light-intensity physical activity and adiposity in childhood. Pediatr Exerc Sci..

[48] Hughes AR, Henderson A, Ortiz-Rodriguez V, Artinou ML, Reilly JJ (2006). Habitual physical activity and sedentary behaviour in a clinical sample of obese children. Int J Obes..

[49] Metallinos-Katsaras ES, Freedson PS, Fulton JE, Sherry B (2007). The association between an objective measure of physical activity and weight status in preschoolers. Obesity..

[50] Carson V, Ridgers ND, Howard BJ, Winkler EA, Healy GN, Owen N (2013). Light-intensity physical activity and cardiometabolic biomarkers in US adolescents. PloS One..

[51] Collings PJ, Brage S, Ridgway CL, Harvey NC, Godfrey KM, Inskip HM (2013). Physical activity intensity, sedentary time, and body composition in preschoolers. Am J Clin Nutr..

[52] Basterfield L, Pearce MS, Adamson AJ, Reilly JK, Parkinson KN, Reilly JJ (2012). Effect of choice of outcome measure on studies of the etiology of obesity in children. Ann Epidemiol..

[53] Biddle S, Mutrie N (2008). Psychology of physical activity: determinants, well-being and interventions.

[54] Talma H, Chinapaw MJ, Bakker B, HiraSing RA, Terwee CB, Altenburg TM (2013). Bioelectrical impedance analysis to estimate body composition in children and adolescents: a systematic review and evidence appraisal of validity, responsiveness, reliability and measurement error. Obes Rev Off J Int Assoc Study Obes..

[55] Corder K, Brage S, Wareham NJ, Ekelund U (2005). Comparison of PAEE from combined and separate heart rate and movement models in children. Med Sci Sports Exerc..

[56] Corder K, Brage S, Mattocks C, Ness A, Riddoch C, Wareham NJ (2007). Comparison of two methods to assess PAEE during six activities in children. Med Sci Sports Exerc..

[57] Thompson D, Batterham AM, Bock S, Robson C, Stokes K (2006). Assessment of low-to-moderate intensity physical activity thermogenesis in young adults using synchronized heart rate and accelerometry with branched-equation modeling. J Nutr..

